# Supplementary material for: Diversity and Correlation Analysis of Endophytes and Top Metabolites in Phlomoides rotata Roots from High-Altitude Habitats
Source: Microorganisms. 2025 Feb 25;13(3):503. doi: 10.3390/microorganisms13030503 (PMC11944690; doi:10.3390/microorganisms13030503)
Supplement: Supplementary file 1 [file microorganisms-13-00503-s001.zip › Tables .pdf]

Table S1 Quality control data for endophytic bacterial sequencing in *P. rotata* roots from 4 habitats

Sample ID	Raw Reads	Clean Reads	Denoised Reads	Merged Reads	Non-chimeric Reads	OTU Number
HN1	160,270	147,211	145,868	135,509	122,193	5,124
HN2	159,665	146,489	145,114	134,350	121,951	5,163
HN3	160,546	146,883	145,645	133,958	119,945	5,085
GL1	159,548	147,256	145,917	135,293	121,147	4,274
GL2	159,630	146,614	145,509	136,964	124,536	4,601
GL3	160,021	147,022	145,705	131,162	112,923	4,454
YS1	159,890	144,125	143,259	136,020	110,603	3,168
YS2	160,053	143,963	143,035	136,612	111,589	2,909
YS3	159,766	143,549	142,700	135,621	110,018	3,071
CD1	160,130	146,828	145,267	127,036	108,451	4,692
CD2	160,097	146,908	145,394	129,549	113,001	4,633
CD3	160,106	146,682	145,132	131,203	117,096	4,638
Total	1,919,722	1,753,530	1,738,545	1,603,277	1,393,453	51,812
Average	159,977	146,128	144,879	133,606	116,121	4,318

Table S2 Quality control data for endophytic fungal sequencing in *P. rotata* roots from 4 habitats

Sample ID	Raw Reads	Clean Reads	Denoised Reads	Merged Reads	Non-chimeric Reads	OTU Number
HN1	159,835	121,238	121,042	119,014	117,278	1,578
HN2	159,999	120,797	120,443	118,023	116,148	1,551
HN3	159,905	129,870	129,370	127,431	125,999	1,523
GL1	160,085	118,357	118,047	116,018	114,970	1,409
GL2	160,250	125,225	124,914	123,422	122,399	1,373
GL3	159,984	116,768	116,362	114,356	113,508	1,437
YS1	159,985	131,278	130,851	128,563	127,538	1,602
YS2	159,784	119,378	118,983	117,147	114,987	1,411
YS3	160,179	122,171	121,839	119,752	118,051	1,364
CD1	160,008	119,074	118,868	117,109	116,322	1,417
CD2	159,817	128,478	128,244	126,264	125,589	1,397
CD3	159,895	130,895	130,480	128,266	126,690	1,468
Total	1,919,726	[illegible]	[illegible]	1,455,365 1,439,479 17,530	1,439,479	17,530
Average	159,977	123,627	123,287	121,280	119,957	1,461

Table S3 Species annotation and relative abundance analysis of endophytic bacteria in *P. rotata* roots from 4 habitats

Sample	Kindom	Phylum	Class	Order	Family	Genus	Species
HN1	1	31	77	204	437	927	1129
HN2	1	28	75	205	443	922	1145
HN3	1	34	79	216	441	939	1133
GL1	2	37	73	202	402	886	1076
GL2	2	30	75	200	417	950	1157
GL3	1	33	77	201	420	917	1091
YS1	1	27	65	184	370	765	894
YS2	1	25	60	170	342	734	840
YS3	2	28	71	181	377	748	850
CD1	1	33	75	196	405	868	1042
CD2	1	25	61	188	401	856	1063
CD3	1	29	70	195	427	881	1074
Total	2	42	105	314	710	1863	2776

Table S4 Species annotation and relative abundance analysis of endophytic fungi in *P. rotata* roots from 4 habitats

Sample	Kindom	Phylum	Class	Order	Family	Genus	Species
HN1	1	13	43	97	212	393	520
HN2	1	12	45	93	212	387	522
HN3	1	12	43	89	206	385	507
GL1	1	13	37	85	199	370	516
GL2	1	14	42	96	210	398	525
GL3	1	12	38	91	194	372	516
YS1	1	13	39	96	208	398	542
YS2	1	14	47	98	205	374	497
YS3	1	12	41	95	200	372	494
CD1	1	12	41	90	202	376	526
CD2	1	12	41	100	214	394	506
CD3	1	14	46	102	201	382	513
Total	1	15	56	144	368	902	1552

Table S5 Metabolite classification in *P. rotata* roots from 4 habitats

#ID	Class I	name
POS_q205	Vitamins	Thiamine Monochloride
NEG_q117	Terpenoids	Feretoside
NEG_q236	Terpenoids	Shanzhiside Methyl Ester
NEG_q243	Sugars and alcohols	Sucrose
NEG_q147	Sugars and alcohols	Isomaltose
NEG_q257	Sugars and alcohols	Turanose
POS_q160	Sugars and alcohols	N-Acetylneuraminic Acid
POS_q125	Sugars and alcohols	Kaempferol-3-O-B-D-Glucosyl(1-2)Rhamnoside
NEG_q262	Polyphenols	Verbascoside
NEG_q68	Polyphenols	Chlorogenic Acid
NEG_q118	Phenylpropanoids	Forsythiaside A
NEG_q119	Phenylpropanoids	Forsythoside B
NEG_q120	Phenylpropanoids	Forsythoside I
NEG_q56	Phenylpropanoids	Angoroside C
POS_q22	Others	4-Methyl-5-Thiazoleethanol
NEG_q171	Organic acid	Malic Acid
NEG_q237	Organic acid	(S)-Malic Acid
NEG_q100	Organic acid	D-(+)-Malic Acid
POS_q9	Nucleotides	2'-Deoxyadenosine
POS_q5	Ketones, Aldehydes, Acids	1-Phenylpentan-1-One
POS_q46	Amino acids	Aurantiamide Acetate
NEG_q163	Amino acids	L-Ornithine (Hydrochloride)

NEG_q22	Amino acids	3-(Carboxymethylamino)Propanoic Acid
POS_q74	Amino acids	Cyclo(Ile-Leu)
NEG_q159	Amino acids	L-Glutamic Acid
NEG_q28	Amino acids	4-Hydroxyisoleucine
NEG_q94	Amino acids	Dl-Arginine
POS_q204	Alkaloids	Stachydrine Hydrochloride
POS_q203	Alkaloids	Stachydrine
POS_q107	Alkaloids	Haplopine
