# Supplementary figures and images for: Diversity and Correlation Analysis of Endophytes and Top Metabolites in Phlomoides rotata Roots from High-Altitude Habitats
Source: Microorganisms. 2025 Feb 25;13(3):503. doi: 10.3390/microorganisms13030503 (PMC11944690; doi:10.3390/microorganisms13030503)

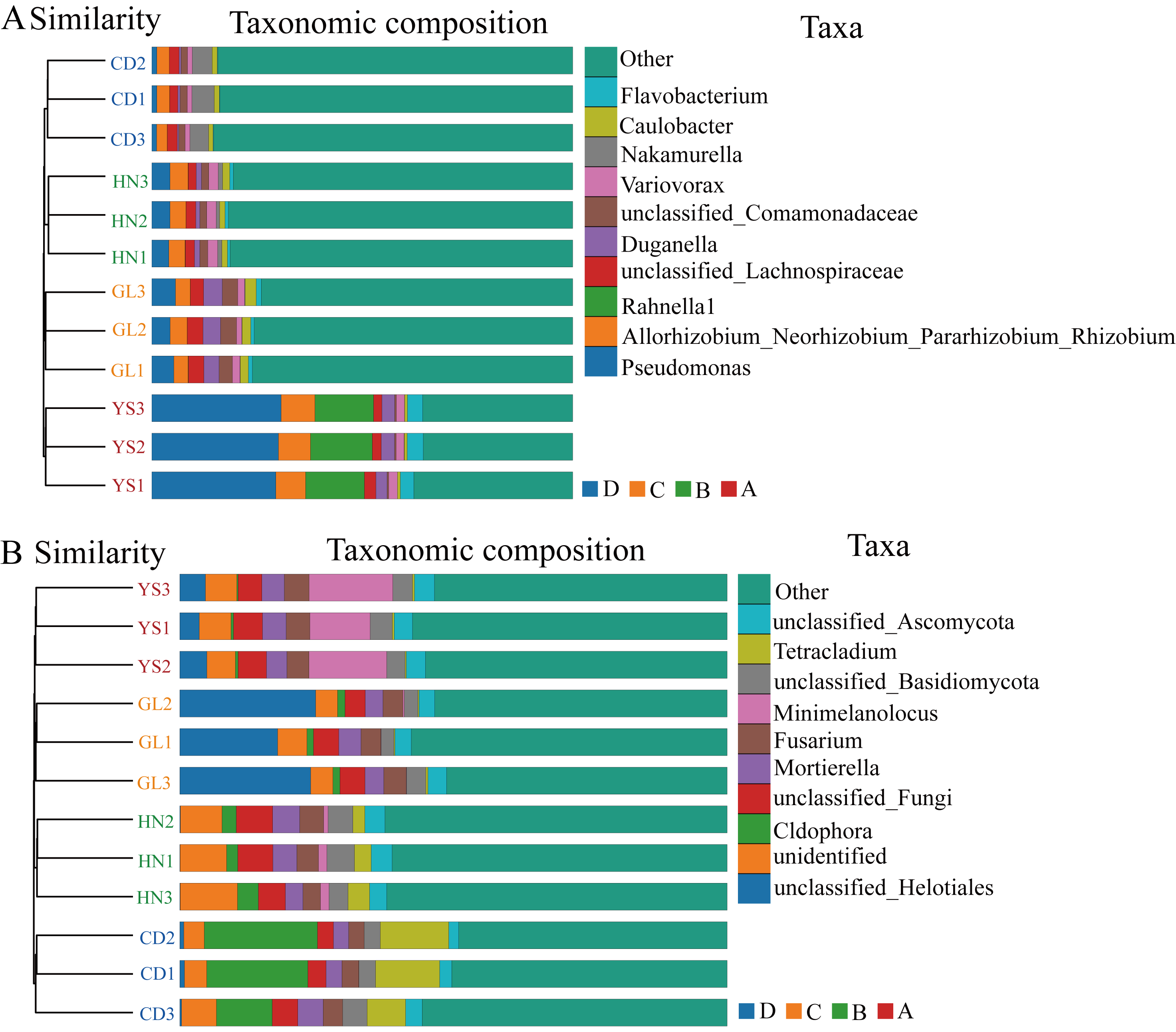

Supplement: Supplementary file 1 [file microorganisms-13-00503-s001.zip › Figure S1.tif]

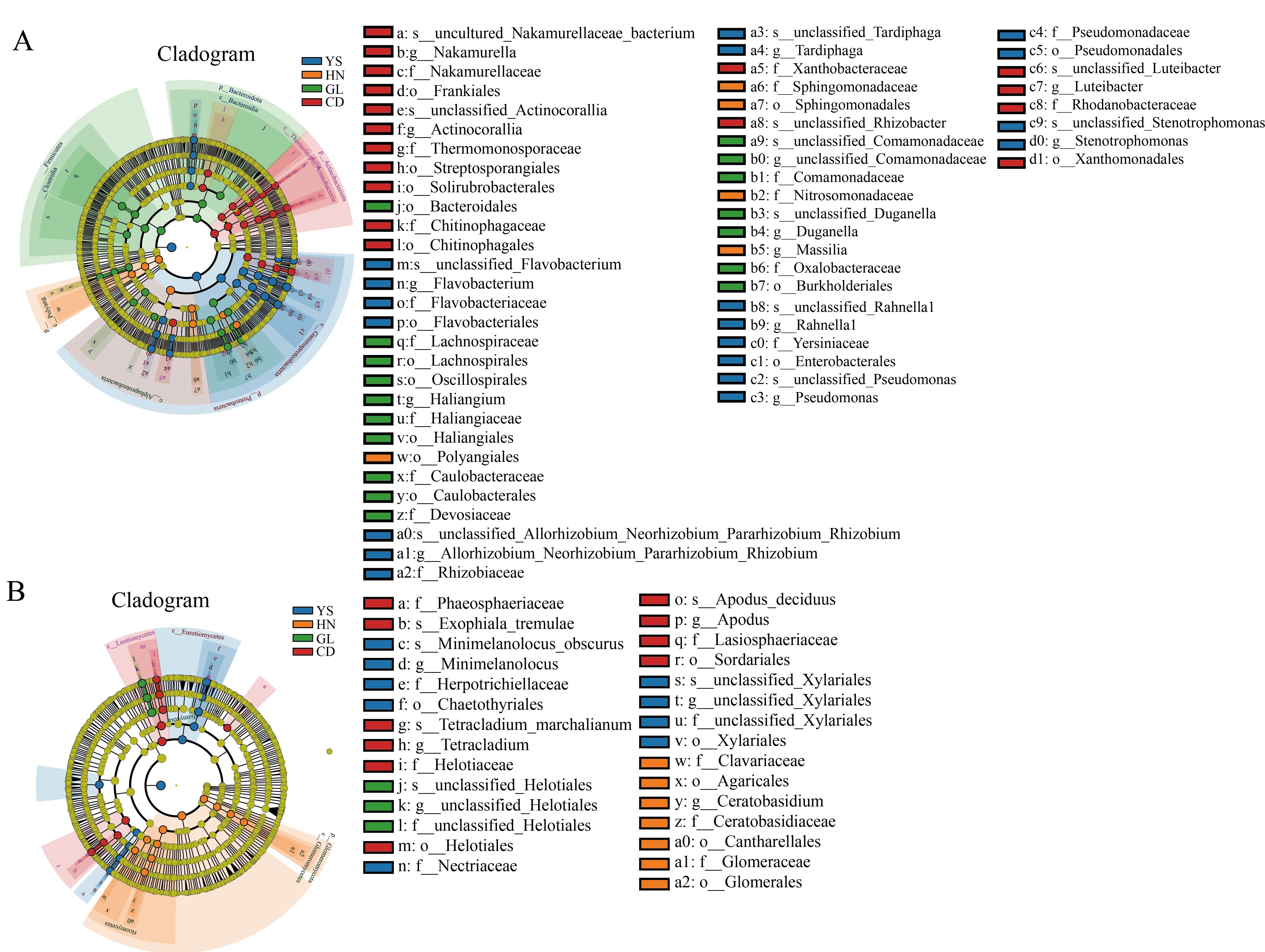

Supplement: Supplementary file 1 [file microorganisms-13-00503-s001.zip › figure S2.tif]
